# Dyslexia Telerehabilitation during the COVID-19 Pandemic: Results of a Rhythm-Based Intervention for Reading

**DOI:** 10.3390/children8111011

**Published:** 2021-11-05

**Authors:** Alice Cancer, Daniela Sarti, Marinella De Salvatore, Elisa Granocchio, Daniela Pia Rosaria Chieffo, Alessandro Antonietti

**Affiliations:** 1 Department of Psychology, Università Cattolica del Sacro Cuore, 20123 Milan, Italy; alessandro.antonietti@unicatt.it; 2 Developmental Neurology Unit—Language and Learning Disorders Service, Fondazione IRCCS Istituto Neurologico Carlo Besta, 20133 Milan, Italy; daniela.sarti@istituto-besta.it (D.S.); marinella.desalvatore@istituto-besta.it (M.D.S.); elisa.granocchio@istituto-besta.it (E.G.); 3 Department of Life Sciences and Public Health, Università Cattolica del Sacro Cuore, 00168 Rome, Italy; danielapiarosaria.chieffo@policlinicogemelli.it

**Keywords:** dyslexia, telerehabilitation, rhythm, music therapy, intervention

## Abstract

The COVID-19 outbreak necessitated a reorganization of the rehabilitation practices for Learning Disorders (LDs). During the lockdown phase, telerehabilitation offered the possibility to continue training interventions while enabling social distancing. Given such an advantage of telerehabilitation methods for LDs, clinical research is still needed to test the effectiveness of diverse teletraining approaches by comparing their outcomes with those of face-to-face interventions. To compare the effectiveness of telerehabilitation vs. in-presence rehabilitation of dyslexia, a rhythm-based intervention for reading, called Rhythmic Reading Training (RRT), was tested in a small-scale clinical trial during the lockdown phase of the COVID-19 pandemic. Thirty children aged 8–13 with a diagnosis of developmental dyslexia were assigned to either a telerehabilitation or an in-presence rehabilitation setting and received RRT for 10 biweekly sessions of 45 min, supervised by a trained practitioner. The results showed that both telerehabilitation and in-presence rehabilitation were effective in improving reading and rapid automatized naming in children with dyslexia and that the effects were comparable between settings. Therefore, RRT was found to be effective in spite of the administration method (remote or in-presence). These results confirm the potential of telemedicine for the rehabilitation of LDs. Clinical Trial ID: NCT04995471.

## 1. Introduction

Telemedicine [1] has made it possible to treat patients in their own environment. Since it started and spread, telemedicine has been proven to be a great asset where no easy access to healthcare was possible. A great number of studies have been conducted in the last decade to explore possibilities given by tech tools in adult/older neuropsychological rehabilitation [2,3,4,5,6]. In recent years, technology has been proven to be a great potential resource for developmental age and attention to tech tools in neurodevelopmental disorder rehabilitation has grown fast. Telerehabilitation represents a great advantage when it comes to developmental age. Several studies investigated the effectiveness of brain computer interfaces, virtual reality tools, and computer-based training for Attention Deficit Disorder (ADD) [7,8], Developmental Coordination Disorder (DCD) [9], Autistic Spectrum Disorder (ASD) [10,11], anxiety disorders [12,13], and eating disorders [14,15].

Literature confirms a series of additional advantages given by the use of technology in child rehabilitation. Web-based approaches result, in fact, in increased enthusiasm and a reduced dropout risk. Additionally, a tech approach to rehabilitation could lead to a better generalization of learning (even if no clear agreement is found in literature on this point). Advances in technology are constantly increasing the number of available tools: Bio-neurofeedback, virtual and augmented reality tools, and computer software ensure high motivation in children attending rehabilitation programs because of the playful environment and the quick reward design.

The year 2020 was a worldwide turning point for telemedicine because of the COVID-19 pandemic. Studies on telemedicine have flourished, supported by the urgent need to treat patients while protecting the healthcare of professionals. Telemedicine represents the first-line tool for clinical settings enabling social distancing [16,17,18]. Today, more than ever, the need for evidence-based healthcare technology is increasing to build a strong and cost-effective telerehabilitation healthcare system [19]. Italy was a first-line trench in facing the pandemic. Children with neurodevelopmental disorders and their families suffered particularly from the lockdown and social isolation that followed. The possibility of continuing rehabilitation treatments online had a central role in preventing psychopathological risks generated by emergency situations in more fragile subjects [20]. Information Computer Technology (ICT) has enabled tailored interventions dedicated to the patient’s individual needs through the use of computers, tablets, and other media (e.g., videocalls and messaging apps).

Several telemedicine projects dedicated to psychological healthcare have been implemented in the last year [18,21] and the urgency for telehealth programs has come to light [22,23,24,25,26]. Similar to other psychological interventions, the rehabilitation practices for Learning Disorders (LDs) had to be reorganized in the context of the COVID-19 outbreak. To prevent possible negative outcomes associated with untreated neurodevelopmental disorders, Italian clinical guidelines encourage the early identification and continuous treatments of LDs to reduce risk factors and comorbidity [27].

An Italian example of the reorganization of LD practices imposed by COVID-19 was recently described in an article by Sarti and colleagues [28]. More precisely, the Language and Learning Disorders Service of the Fondazione IRCCS Istituto Neurologico Carlo Besta of Milan, Italy [29] reformulated the children’s rehabilitation plan by integrating telemedicine in order to ensure continuity of care for patients. The reorganization involved children with Specific Learning Disorders (SLDs) and preschoolers with Language and Speech Disorders. To select the appropriate patients for telerehabilitation, some feasibility criteria were followed: neuropsychological criteria, attentional, adaptive, and motivational requirements of children as well as network requirements such as connection stability, availability of devices in the family, and the children’s and their families’ familiarity and autonomy with new technologies. In this regard, the level of competence of children with LDs should not be overestimated, as recent studies have shown that, contrary to expectations, Italian students show little competence in the use of ICT [30].

As for the specific case of reading intervention in developmental dyslexia (DD), several telerehabilitation programs were already available in Italy since before the pandemic. Such programs are based on different developmental reading models. For instance, the online software Reading Trainer [31,32], based on the dual-route developmental reading model [33,34], is a sublexical and lexical treatment aimed to facilitate the correspondences between graphemes and phonemes and to automatize the naming of sublexical units (i.e., syllables and morphemes) and words. Another homebased software is Run the RAN [31,32], a process-oriented intervention addressing difficulties in rapid automatized naming (RAN) tasks, which is one of the main cognitive deficits underlying dyslexia and strongly relates to reading fluency. Run the RAN requires the child to name timed visual nonalphanumeric stimuli (i.e., colors or pictures) as quickly as possible. To remotely improve reading efficiency in children with DD, another group of Italian researchers designed Tachidino [35], a telerehabilitation method based both on hemisphere-specific stimulation, following Bakker’s Balance Model [36,37], and on visuospatial selective attention training and the ability to manage visual crowding, according to the magnocellular deficit theory of DD [38,39]. The Tachidino web application includes a large library of Italian words, categorized on the basis of their morpho-linguistic characteristics and specific reading strategies, which are presented tachistoscopically.

The aim of the present study was to compare the effectiveness of telerehabilitation vs. in-presence rehabilitation of reading disorders using a rhythm-based intervention for reading (i.e., Rhythmic Reading Training, RRT).

Extended literature posited the possibility of improving reading abilities in students with DD using music and auditory-based training (for a review, see [40]). Specifically, music activities are supposed to address the phonological difficulties underlying DD through an improved ability to process the temporal components of acoustic stimuli and, ultimately, to improve rhythmic and synchronization abilities. The rhythmic processing of speech cues is indeed fundamental for phonological and reading development in children [41,42,43], and it is significantly impaired in dyslexia [41,42,44]. A recent literature review by Cancer and Antonietti [40] reported that music-based and auditory-based interventions for dyslexia produced significant effects on phonological abilities, thus supporting the hypothesis of a transfer effect of music training on phonological and reading skills.

Among the reviewed effective interventions, RRT, a rhythm-based training software, was designed to improve reading in Italian students with DD [45,46,47]. The program includes beat-based reading exercises, in which the reader has to synchronize his/her speech with that of an isochronous beat with an increasing pace. Such an approach was developed to facilitate the segmentation of the phonological units mapped into the metric structure of language by stressing each syllable onset sound and, simultaneously, improving synchronization skills.

The efficacy of RRT was previously tested in several controlled clinical trials, in which the rehabilitation method was delivered to children and adolescents with DD under the supervision of an expert practitioner for 10–20 sessions. More precisely, RRT was found to be effective in improving reading speed and accuracy compared to spontaneous reading development [48]. Furthermore, RRT’s efficacy was found to be comparable to that of a traditional reading intervention involving homework [49] and to that of a novel intervention involving visuo-spatial and attentional stimulations [50], with specific larger effects of RRT on pseudo-word reading speed. Finally, significant improvements following RRT were maintained three months after completion of the intervention [46]. Such evidence confirms the feasibility and efficacy of the RRT method for reading training in DD.

Previous applications of RRT were performed in face-to-face settings. To test the possibility of administering RRT remotely, we conducted a small-scale investigation during the lockdown phase of the COVID-19 pandemic. More precisely, we explored the feasibility and effects of RRT remote administration by comparing it with the traditional face-to-face RRT setting.

## 2. Materials and Methods

### 2.1. Participants

Children with a specific reading disorder, who had previously received a diagnosis of developmental dyslexia (ICD-10 code: F81.0) [51] based on the LD diagnostic procedure adopted in Italy [52], were recruited from patients of the LD Services of three Italian clinical institutions. Eligibility of participants was determined according to the following inclusion criteria: children between the ages of 8 and 14with a reading performance of > 2 SD below the norm in at least one standardized reading test, normal intelligence (TIQ ≥ 80), and the absence of psychiatric and/or neurological conditions.

Thirty children aged between 8 and 13 years (M = 9.89; SD = 1.31; 12 females), who were attending the 2nd to 7th grades, met the inclusion criteria and were included in the study. Parents’ written informed consent was obtained prior the recruitment.

### 2.2. Procedure

The trial protocol was registered on Clinicaltrials.gov (Clinical Trial ID: NCT04995471). Participants were divided into two homogeneous subgroups of the same size (*n* = 15) using stratified sampling by matching participant for age, sex, TIQ, and reading baseline performance. Each subgroup was then assigned to one of two intervention conditions: telerehabilitation vs. in-presence rehabilitation. Both subgroups received RRT for 10 biweekly sessions of approximately 45 min, supervised by a trained RRT practitioner for a total of 7.5 h of intervention.

To compare the telerehabilitation vs. in-presence methods, a battery of standardized tests, namely, reading and rapid automatized naming tests, were administered to participants before (pre) and immediately after (post) training.

### 2.3. Intervention Conditions

RRT is a computerized training program that includes music-based reading exercises. RRT’s activities were designed to address multiple reading subprocesses, specifically syllabic blending, syllabic reading, word recognition, and sublexical decoding [45]. All exercises include a simple rhythmic-melodic stimulation coordinated with a visual cue. The sequential visual selection of each verbal stimuli is synchronized with a regular beat, whose speed is set by the practitioner based on each participant’s initial reading level. Speed and difficulty of the exercises are adjusted by the trainer during each session. Practitioners are instructed to gradually increase the speed settings within each activity when the participant reaches a 90% accuracy rate in the previous reading performance. Reading accuracy is assessed by the practitioner during training by counting reading errors in each exercise.

As regards the in-presence administration of RRT, the training sessions took place in a quiet room, where the practitioner and the child were seated next to each other in front of the same computer screen.

In the telerehabilitation setting, RRT was administered using the sharing screen feature of a video teleconferencing platform (i.e., Microsoft Teams). The trainer ran the RRT software on their computer and shared their screen with the participant during a conference call. Prior to the beginning of the intervention, the trainer planned a test call with the participant to check the stability of their internet connection and the quality of the screen sharing procedure.

### 2.4. Measures

Participants’ clinical documentation was collected and information about their medical history, diagnosis, and intellectual functioning measures (i.e., Total IQ derived from the Wechsler Intelligence Scale for Children—Fourth Edition [53]) was retrieved from it.

Reading abilities were assessed using standardized tests providing accuracy and speed scores. More precisely, the ability to read aloud age-normed text passages was measured using the ‘New MT reading tests for junior high school’ [54]. Furthermore, the ‘Assessment Battery for Developmental Reading and Spelling Disorders-2′ [55] was used to assess word and pseudo-word reading (i.e., 4 lists of 28 words with different lengths and frequency of use; 2 lists of 16 pseudo-words with different lengths).

Finally, rapid automatized naming (RAN) was assessed using the ‘Rapid Automatized Naming Test (RAN)—Figures’ Test [56], in which participants were required to rapidly and sequentially name a series of black and white figures (i.e., pear, train, dog, star, hand) presented in two 10 × 5 matrixes. RAN speed (i.e., seconds) was recorded.

### 2.5. Statistical Analyses

A sample size of 30 was calculated to be enough to achieve a power of 0.80 to detect a medium effect size (η^2^ = 0.06) in a mixed factorial ANOVA 2 × 2—the model which we planned to carry out to test our hypothesis—setting alpha at 0.05.

Assumptions of normality (Kolmogorov–Smirnov ps ranging between 0.25 and 0.99) and homogeneity of error variance (Levene’s ps ranging between 0.08 and 0.99) were met for all outcome variables. Therefore, we opted for parametric comparisons (GLMs).

First, we checked that the stratified sampling methods produced homogenous subgroups for age, sex, and baseline reading abilities using the Chi-squared test for categorical variables and independent samples t-tests for continuous variables.

Then, a mixed factorial ANOVA 2 × 2 (Condition: telerehabilitation vs. in-presence; Phase: pre vs. post) was tested for each outcome variable (i.e., reading speed, reading accuracy, RAN). As for the reading measures, composite speed and accuracy scores were computed by averaging text, word, and pseudo-word z-scores.

## 3. Results

The participants’ characteristics are reported in Table 1. Age (t (26) = 0.10; *p* = 0.92), TIQ (t (25) = 0.10; *p* = 0.94), sex (χ^2^ = 2.22; *p* = 0.14), and school grade (χ^2^ = 8.03; *p* = 0.15) did not differ between groups. In addition, no difference was found between groups in pre-training composite reading measures (reading speed: t (28) = 0.44; *p* = 0.66; reading accuracy: t (28) = 0.19; *p* = 0.85).

Descriptive statistics of the pre-training and post-training scores for each intervention condition are reported in Table 2.

Reading speed and reading accuracy improved after training in both conditions, as confirmed by significant Phase main effects (reading speed: F(1,28) = 70.58; *p* < 0.001; η^2^ = 0.11; reading accuracy: F(1,28) = 4.55; *p* < 0.04; η^2^ = 0.02). Conversely, the Phase × Condition interaction effect was nonsignificant for both reading outcomes (reading speed: F(1,28) = 0.09; *p* = 0.77; reading accuracy: F(1,28) = 0.84; *p* = 0.37), thus showing no telerehabilitation vs. in-presence rehabilitation difference.

As for the secondary outcome measure, similar results were found for RAN speed, with a significant Phase main effect (F(1,28) = 5.45; *p* < 0.04; η^2^ = 0.07) and a nonsignificant Phase × Condition interaction effect (F(1,28) = 0.67; *p* = 0.43).

## 4. Discussion and Conclusions

During the unprecedented events associated with the spread of the COVID-19 disease, telemedicine constituted a chance to treat neurodevelopmental disorders through tailored and goal-oriented interventions, while maintaining social distancing. Given the evident advantages of telerehabilitation methods, clinical research is needed to test their effectiveness in the intervention of LDs, such as DD, by comparing the outcomes with those of traditional face-to-face interventions.

Previous Italian literature analyzed the efficacy of several telerehabilitation methods for DD, showing promising results. In a study that included 34 children with DD attending primary or secondary schools, Tucci and colleagues [57] confirmed the efficacy of an intervention using the online software Reading Trainer [31,32]. After a training period of approximately 13 weeks, with 15 min training sessions at least 3 times a week, the authors found a significant improvement of reading fluency and accuracy. Another study by Pecini and colleagues [32] compared the effects of the Run the RAN training with those of the Reading Trainer, administered in 5–15 min sessions for a total of 8 and 12 h respectively in a group of 45 children with DD. The results showed that the reading speed and accuracy improved regardless of the training type and that improvements lasted for 3 months after the end of the intervention.

Although there were significant within-group effects of telerehabilitation, these studies did not include an in-presence control condition. To our knowledge, the only Italian study which compared telerehabilitation and in-presence rehabilitation of DD is a recently published study by Lorusso and colleagues [35]. The authors compared 65 children with DD who underwent a remote intervention using the Tachidino web application with 49 children who received an in-presence visuospatial intervention (i.e., Action Video Games training [58] in combination with Visual Hemispheric Specific Stimulation [39]). The results showed that improvements emerged in both groups in terms of both speed and accuracy of reading.

The present small-scale clinical trial is the first attempt to measure the specific effect of the online remote administration of an Italian reading training by comparing parallel training conditions with matching materials, activities, procedures, and training schedules. The results of the present study showed that both telerehabilitation and in-presence rehabilitation of reading abilities using a rhythm-based computerized intervention were effective in improving reading and RAN abilities in students with DD and that the effects were comparable between settings. Such results demonstrate that RRT is effective in spite of the administration method (remote or in-presence), thus adding evidence of its potential as a rehabilitation method for DD.

In comparison with other telerehabilitation methods, the RRT makes it possible to achieve significant progress in a shorter period of time, namely, 7.5 h. In addition, the need for support by the parent/caregiver appears to be less significant compared to other methods (e.g., reading errors were recorded by the online trainer during rehabilitation sessions, and no parental supervision was necessary).

Furthermore, these results confirmed the telemedicine potential for the rehabilitation of LDs, which was previously highlighted in several studies conducted during the COVID-19 pandemic. Numerous papers reported on the results obtained in telemedicine satisfaction questionnaires that were completed by adult patients and children’s caregivers and that highlighted an overall good level of satisfaction concerning the remodeling of services [29]. Telerehabilitation for LDs has made it possible to maintain therapeutic continuity for younger patients. In particular, the easy remote adaptability of the RRT was a strong point during a period that required rapidity/timeliness in changes within the rehabilitation practices settings [59]. Concerning the reception of the rhythm-based intervention, spontaneous comments by participants revealed that children generally accepted the teletraining positively, showing curiosity towards RRT and expressing their satisfaction with regards to the results obtained. Consistently, parents expressed overall satisfaction with regard to maintaining an improving trend with their children, as anecdotally recorded, given the concerns that families of children with neurodevelopmental disorders had during the pandemic period [60].

As regards telerehabilitation, the remote setting was characterized by both strengths and limitations. On the one hand, the home context where the rehabilitation took place was perceived as reassuring and comfortable for children. On the other hand, the relational components of rehabilitation were less controllable compared to face-to-face interactions. In some cases, the unfamiliarity with new technologies did represent a limit, since children were not autonomous and parents were required to help them. However, this occurred more frequently at the beginning of the remote training path and most children learned quite early to manage the telerehabilitation setting autonomously. We suggest that future investigations should include a preintervention tech training for children who may struggle with the use of technology. Furthermore, for the most fragile patients, such as younger children, children with more severe reading difficulties, and a reduced attention span, the teletraining was more tiring and several interruptions were required during the sessions. As for the other studies presented on the telerehabilitation of DD in regular orthography, the present study confirms that homebased software may foster the automatization of the reading processes after only a few months of teletraining.

Besides the small sample size, one of the limitations of the present study was the lack of follow-up measures, which would assess the long-term effects of the intervention. However, a previous study on RRT showed that reading gains after intervention lasted for three months [46]. Although we expect that such results should be replicated in both in-presence and telerehabilitation settings, future investigations may compare the long-term effects of RRT in different administration conditions.

Finally, despite the positive outcomes of telerehabilitation, as shown by the results of the present study, we believe that reading rehabilitation is better administered in a presence-remote hybrid setting. While telemedicine can facilitate the rehabilitation of LDs in certain conditions, for the most fragile patients, face-to-face treatment is preferable due to the irreplaceable interpersonal variables within the clinical relationship.

## Figures and Tables

**Table 1:** Participants’ characteristics.

	Telerehabilitation	In-Presence Rehabilitation
2nd grade ^1^	0	1
3rd grade ^1^	2	8
4th grade ^1^	5	2
5th grade ^1^	4	3
6th grade ^1^	3	1
7th grade ^1^	1	0
Age ^2^	10.30 (1.38)	9.49 (1.16)
Male ^1^	7	11
Female ^1^	8	4
TIQ ^2,3^	98.20 (9.42)	97.85 (15.01)
Baseline reading speed ^2,4^	−1.70 (0.63)	−1.80 (0.62)
Baseline reading accuracy ^2,4^	−2.89 (2.06)	−3.35 (3.94)

^1^ Counts. ^2^ Mean (Standard Deviations). ^3^ Total IQ composite score derived from the Wechsler Intelligence Scale for Children—Fourth Edition. ^4^ Z-scores.

**Table 2:** Descriptive statistics of reading and RAN speed pretraining and post-training z-scores, for each rehabilitation condition (i.e., telerehabilitation vs. in-presence rehabilitation).

Condition	Parameter	Phase	M (SD)
Telerehabilitation	Reading speed	Pre	−1.70 (0.63)
		Post	−1.21 (0.72)
	Reading accuracy	Pre	−2.89 (2.06)
		Post	−2.47 (2.42)
	RAN	Pre	−1.82 (1.18)
		Post	−1.19 (1.86)
In-presence rehabilitation	Reading speed	Pre	−1.80 (0.62)
		Post	−1.34 (0.83)
	Reading accuracy	Pre	−3.35 (3.94)
		Post	−2.30 (2.29)
	RAN	Pre	−3.02 (2.69)
		Post	−1.71 (1.14)

## Data Availability

The data presented in this study are available on request from the corresponding author.
